# Donor and recipient hematopoietic stem and progenitor cells mobilization in liver transplantation patients

**DOI:** 10.1186/s13287-024-03855-5

**Published:** 2024-07-29

**Authors:** Yao Zhi, Wei Qiu, Guangyao Tian, Shifei Song, Wenchao Zhao, Xiaodong Du, Xiaodong Sun, Yuguo Chen, Heyu Huang, Jing Li, Ying Yu, Mingqian Li, Guoyue Lv

**Affiliations:** Department of Hepatobiliary and Pancreatic Surgery, General Surgery Center, The First Hospital of Jilin University, Changchun, 130021 China

**Keywords:** Hematopoietic stem and progenitor cells, Alloimmune response, Liver transplantation, Hematopoietic stem and progenitor cells mobilization

## Abstract

**Background:**

Hematopoietic stem and progenitor cells (HSPCs) mobilize from bone marrow to peripheral blood in response to stress. The impact of alloresponse-induced stress on HSPCs mobilization in human liver transplantation (LTx) recipients remains under-investigated.

**Methods:**

Peripheral blood mononuclear cell (PBMC) samples were longitudinally collected from pre- to post-LTx for one year from 36 recipients with acute rejection (AR), 74 recipients without rejection (NR), and 5 recipients with graft-versus-host disease (GVHD). 28 PBMC samples from age-matched healthy donors were collected as healthy control (HC). Multi-color flow cytometry (MCFC) was used to immunophenotype HSPCs and their subpopulations. Donor recipient-distinguishable major histocompatibility complex (MHC) antibodies determined cell origin.

**Results:**

Before LTx, patients who developed AR after transplant contained more HSPCs in PBMC samples than HC, while the NR group patients contained fewer HSPCs than HC. After LTx, the HSPC ratio in the AR group sharply decreased and became less than HC within six months, and dropped to a comparable NR level afterward. During the one-year follow-up period, myeloid progenitors (MPs) biased differentiation was observed in all LTx recipients who were under tacrolimus-based immunosuppressive treatment. During both AR and GVHD episodes, the recipient-derived and donor-derived HSPCs mobilized into the recipient’s blood-circulation and migrated to the target tissue, respectively. The HSPCs percentage in blood reduced after the disease was cured.

**Conclusions:**

A preoperative high HSPC ratio in blood characterizes recipients who developed AR after LTx. Recipients exhibited a decline in blood-circulating HSPCs after transplant, the cells mobilized into the blood and migrated to target tissue during alloresponse.

**Supplementary Information:**

The online version contains supplementary material available at 10.1186/s13287-024-03855-5.

## Background

Liver transplantation (LTx) has been established as an essential clinical option in the treatment of patients with end-stage liver failure [1]. Alloresponse is an immune reaction towards allogeneic antigens, it plays a central role in both acute rejection (AR) and graft versus host disease (GVHD) following LTx to hinder the success of LTx [2]. AR occurs in about 30% of LTx patients on tacrolimus-based immunosuppressive protocols, it increases the risk of graft failure, all-cause mortality, and graft failure-related death by damaging the liver graft [3]. Despite the rarity, GVHD is a deadly immune complication following LTx that severely damages several of the recipient’s target tissues including skin, gastrointestinal tract and hematopoietic tissues [4, 5]. Both of the inflammatory processes trigger stress responses in the transplanted recipients.

Hematopoietic stem and progenitor cells (HSPCs) possess the ability of self-renewal and multi-lineage differentiation to sustain hematopoietic homeostasis. Typically, most of the HSPCs reside in the bone marrow, with only a tiny portion circulating in peripheral blood and tissues. Under emergency stress, hematopoietic stem cells (HSCs) are activated and proliferate, a phenomenon known as emergency myelopoiesis [6, 7]. Several studies have reported that stress-induced conditions, such as infection and inflammation, can induce the mobilization of HSPCs. During this process, HSPCs exhibit the capacity to discern signals indicative of inflammation to mobilize and accumulate within peripheral tissues that are subject to inflammatory processes to contribute to the enhancement of the immune cell population, thereby boosting the tissue’s defensive capabilities against infection [8–10]. However, the HSPC mobilization before and after LTx and during an AR or GVHD episode in LTx recipients has been inadequately explored.

We longitudinally monitor the HSPCs and their subpopulation composition to investigate the dynamics and differentiation patterns of HSPCs in AR patients, GVHD patients, and non-rejection (NR) recipients. HSPCs were divided into 5 subpopulations, including HSCs, multi-potent progenitors (MPPs), lymphoid-primed multipotent progenitors (LMPPs), common lymphoid progenitors (CLPs), and myeloid progenitors (MPs) according to their surface markers expression [11]. First, compared with the age-matched healthy donors, we investigated the preoperative HSPCs proportion and their subset distribution characterization in the peripheral blood of LTx patients. We also delineated the dynamics of donor or recipient HSPCs in blood after transplant during the one-year follow-up period, and the changes to the alloresponse in an AR or GVHD episode.

## Methods

### Study population

**Fig. 1:**
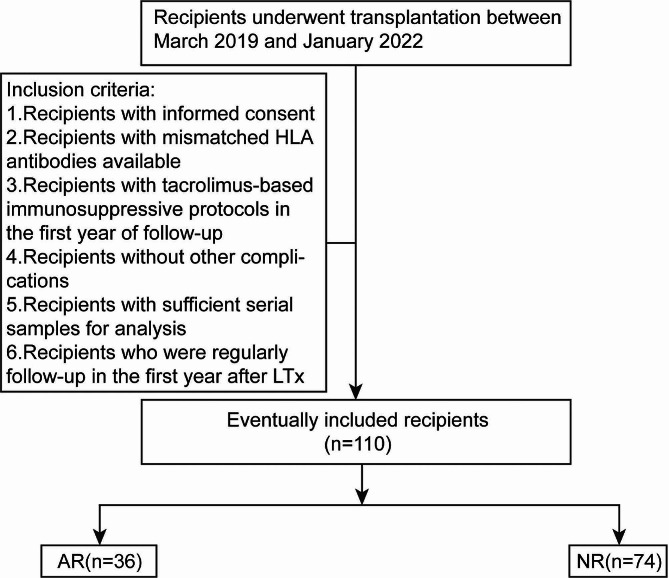
Flowchart of the study design. *Abbreviations* Acute rejection (AR), graft-versus-host disease (GVHD), human leukocyte antigen (HLA), Healthy control (HC) recipients without rejection (NR)

All recipients’ specimens were retrospectively collected from the Department of Biobank, Division of Clinical Research, the First Hospital of Jilin University. The HLA typing information of both recipients and donors were routinely tested before LTx in our liver transplantation center. We selected blood samples from patients who underwent LTx between March 2019 and January 2022. Inclusion criteria were as follows: (1) recipients with informed consent, (2) recipients with mismatched human leukocyte antigen (HLA) antibodies available to allow accurate determination of cell origin using HLA antibodies by flow cytometry (the HLA antibody sensitivity was tested by cell dilution assays of artificial mixtures of known ratios of donor and recipient pretransplant cells from 0.8 to 50% [12] as shown in Figure S1A), (3) recipients under tacrolimus-based immunosuppressive protocols in the first year of follow-up, (4) recipients without other complications (such as pathologically diagnosed drug-induced hepatic injury and biliary complications, severe virus infection, tumor recurrence, undiagnosed abnormal liver function) (5) recipients who were regularly follow-up in the first year after LTx and had sufficient serial samples for analysis at the time point before transplantation and on postoperative day (POD) 7 ± 4, POD 21 ± 7, POD 60 ± 21, POD 120 ± 21, POD 180 ± 21, POD 270 ± 30, POD 360 ± 30, as well as during AR or GVHD episode. Peripheral blood mononuclear cells (PBMC) samples from 74 patients with no rejection episode were enrolled in the non-rejection group (NR), and PBMC samples from 36 patients with at least one episode of AR were enrolled in the rejection group (AR) (Fig. 1). A hepatic mononuclear cell (HMC) sample from an explanted liver of secondary LTx from a patient in AR group who developed into chronic rejection after AR was collected.

Basiliximab was administered for induction immunosuppressive therapy on LTx day and POD 4. Post-reperfusion during LTx, two high-dose methylprednisolone boluses (500 mg) were given intravenously. Maintenance immunosuppressive therapy included tacrolimus, mycophenolate mofetil, and prednisone.

AR diagnosis was determined by clinical, biochemical (persistent transaminase and/or bilirubin levels at least twice the upper normal limit without vascular and biliary complications or infection), and histological criteria following the Banff scheme. When AR was confirmed, immunosuppressant and glucocorticoid usage was increased. For mild rejection, patients were treated with oral prednisolone, starting at a dosage of 0.5-1.0 mg/kg/day. For moderate to severe rejection, patients should receive treatment with intravenous methylprednisolone at a dosage of 1–2 mg/kg/day.

Five recipients diagnosed with GVHD between March 2019 and September 2023 were enrolled in the study to illustrate the role of HSPCs in rejection toward recipients. GVHD diagnosis and treatment were conducted as previously reported [13]. Four of the patients resulted in GVHD-related mortality, and one patient resulted in lung infection-related mortality after one month of GVHD was cured. No GVHD patient was followed up for one year. PBMC samples were collected from the five patients, and bone marrow mononuclear cell (BMMC) samples were collected after for-cause bone marrow aspiration during GVHD from two of the five patients.

We also collected PBMC samples from 28 age-matched donors (45 to 60 years old) as HC.

This study was approved by the Ethics Committee of the First Hospital of Jilin University (2023-KS-311).

### PBMC, HMC, BMMC isolation and cryopreservation

#### PBMC isolation

PBMC were isolated from the peripheral blood samples collected in vacutainer tubes containing K2 ethylene diamine tetra acetic acid (BD, USA) by density gradient centrifugation using Lymphoprep (STEMCELL Technologies, Germany).

### HMC isolation

The explanted liver was cut into 3–4 mm pieces and incubated in a pre-warmed digestion buffer, containing 1 mg/ml Collagenase II (Gibco, Thermo Fisher Scientific, USA), 1 mg/ml Collagenase IV (Gibco, Thermo Fisher Scientific, USA), 50 µg/ml DNase I DNase I (Sigma-Aldrich, USA), with rotation at 37 °C for 30 min. Hepatocytes were depleted by 50×g centrifuge. Non-parenchymal cells were then resuspended in 35% Percoll solution (GE Healthcare, USA) and carefully layered on top of the 75% Percoll solution. HMC were subsequently isolated by density gradient centrifugation.

#### BMMC isolation

Bone marrow aspiration samples collected in sodium heparin-containing vacuum tubes (BD, USA) were diluted 4 times with RPMI1640, the BMMCs were subsequently isolated by gradient density centrifugation with Lymphoprep.

#### Cryopreservation

After isolation, cells were either directly analyzed or cryopreserved in NutriFreez^®^D10 Cryopreservation medium (Biological Industries, Israel) and stored in liquid nitrogen for subsequent measurements.

### Multi-color flow cytometry (MCFC) detection

Approximately (1–2)×10^6^ cells were suspended in 100 ml of phosphate-buffered saline (PBS), incubated with antibodies at 4 °C for 30 min, and washed twice with PBS. 7-AAD was added to the samples 10 min before measurement to exclude dead cells. Surface staining of cells was performed using fluorescence-conjugated antibody labels (see Table S2 for a complete list of antibodies). The donor chimerism ratio was determined based on HLA typing information using antibodies against HLA class I mismatched alleles between donor and recipient. Candidate monoclonal antibodies specific to HLA class I alleles were screened to ensure the ability to distinguish donor and recipient cells using pre-LTx PBMC samples in combination with pan-HLA-ABC antibodies as previously described [12]. Stained samples were detected by an LSR Fortessa flow cytometer (BD Biosciences, San Jose, California) or Cytek^®^ Aurora (Cytek^®^ Biosciences, Fremont, California) and analyzed using Flowjo 10.6.2 (TreeStar, Inc., Ashland, OR).

The HSPCs population was defined as CD45^+^LIN (CD3 CD19 CD56 CD14)^−^ CD34^+^. This population was further divided into five subsets: HSC (CD34^+^CD38^−^CD90^+^CD45RA^−^), MPP (CD34^+^CD38^−^CD90^−^CD45RA^−^), LMPP (CD34^+^CD38^−^CD90^−^CD45RA^+^), CLP (CD34^+^CD38^+^CD10^+^), MP (CD34^+^CD38^+^CD10^−^). Gating strategy is shown in Figure S1B.

### Statistical analysis

Statistical analysis was performed using R software v4.2.2 (The R Foundation, Auckland, New Zealand). Continuous variables are presented as means ± SEM and categorical variables as percentages. The chi-square test compared categorical variables. Normally distributed data was analyzed by two-tailed Student’s t-tests and non-parametric data by Mann-Whitney U test. Multiple groups were analyzed using one-way ANOVA with Tukey’s post-tests for normally distributed data or with Kruskal-Wallis with Tukey’s post-tests for non-parametric data. Receiver operating characteristic curve (ROC) analysis was used to obtain optimal sensitivity and specificity of the studied markers. Paired t-tests and one-way ANOVA were used to analyze individuals at different time points. The correlation analysis was conducted using the Pearson correlation test method. *P* value < 0.05 was considered statistically significant. All graphics were compiled by Adobe Illustrator CC 2021 (Ventura, CA).

## Results

### Patient characteristics

The characteristics of recipients in the AR group and NR group are displayed in Table 1. 74.5% of recipients were more than 45 years old at the time of transplantation and 79.1% were male recipients. The most indications for LTx were HBV-related cirrhosis (57.3%). The AR group contained fewer patients over 45 years old than the NR group (*P* = 0.013).

**Table 1:** Characteristics of the liver transplant recipients included in the study

	AR (36)	NR (74)	*P*-value
BMI (kg/m^2^)	26.11 ± 19.74	23.19 ± 4.38	0.226
Recipient gender (%)			0.627
Male	27 (75.0)	60 (81.1)	
Female	9 (25.0)	14 (18.9)	
Donor gender (%)			1.000
Male	28 (77.8)	59 (79.7)	
Female	8 (22.2)	15 (20.3)	
Recipient Age (%)			0.013
≥ 45	21 (58.3)	61 (82.4)	
<45	15 (41.7)	13 (17.6)	
Donor Age (%)			0.076
≥ 45	30 (83.3)	48 (64.9)	
<45	6 (16.7)	26 (35.1)	
Donor source			0.116
Living liver donation	2 (5.6)	2 (2.7)	
Cerebrovascular disease	29 (80.6)	48 (64.9)	
Cerebral trauma	5 (13.9)	24 (32.4)	
Main indications for LTx (%)			0.514
Alcohol-related cirrhosis	4 (11.1)	11 (14.9)	
Hepatitis B-related cirrhosis	19 (52.8)	44 (59.4)	
Hepatitis C-related cirrhosis	2 (5.6)	6 (8.1)	
Both Hepatitis B- and Hepatitis C-related cirrhosis	0 (0)	3 (4.1)	
Other	11 (30.5)	10 (13.5)	
Presence of hepatocellular carcinoma (%)	10 (27.8)	21 (26.8)	1.000
cold ischemia time (min)	398 ± 126.07	351.30 ± 140.13	0.097
HLA mismatches (A + B)	2.19 ± 0.75	2.35 ± 0.67	0.271

We also enrolled 5 patients with post-liver transplant GVHD. The characteristics of GVHD patients are displayed in Table 2. Considering the few subject numbers in this group, statistical differences in patient characteristics between GVHD patients with other patients were not calculated.

**Table 2:** Characteristics of the GVHD recipients

	PT062	PT143	PT439	PT455	PT499
Recipient gender	Male	Male	Male	Male	Male
Donor gender	Female	Male	Male	Male	Male
Recipient age	54	43	56	30	62
Donor age	48	55	49	58	48
Donor source	Cerebral trauma	Cerebrovascular disease	Cerebrovascular disease	Cerebrovascular disease	Cerebrovascular disease
Indications for LTx	Hepatitis B-related cirrhosis	Hepatitis B-related cirrhosis, liver cancer	Hepatitis B-related cirrhosis, liver cancer	Hepatitis B-related cirrhosis	liver cancer
Recipient HLA type	A2,-, B62,75,DR4,8,DQ8,6	A11,29, B7,13, DR4,12, DQ7,-	A2,24, B62,75, DR9,15, DQ9,6	A1,31, BW51,54, DR9,16, DQ9,5	A2,33, B71,54, DR4,12, DQ7,-
Donor HLA type	A11,30, B13,75, DR7,15, DQ6,2	A1,24, B48,57, DR4,7, DQ8,9	A1,30, B62,53, DR4,15, DQ8,6	A24,30, B64,54, DR8,15, DQ6,-	A11,26, B71,39, DR14,-, DQ5,-
Diagnosis (POD)	60	32	44	19	21
Outcomes	Died	Cured	Died	Died	Died

### HSPCs were enriched within AR recipients’ blood before LTx

**Fig. 2:**
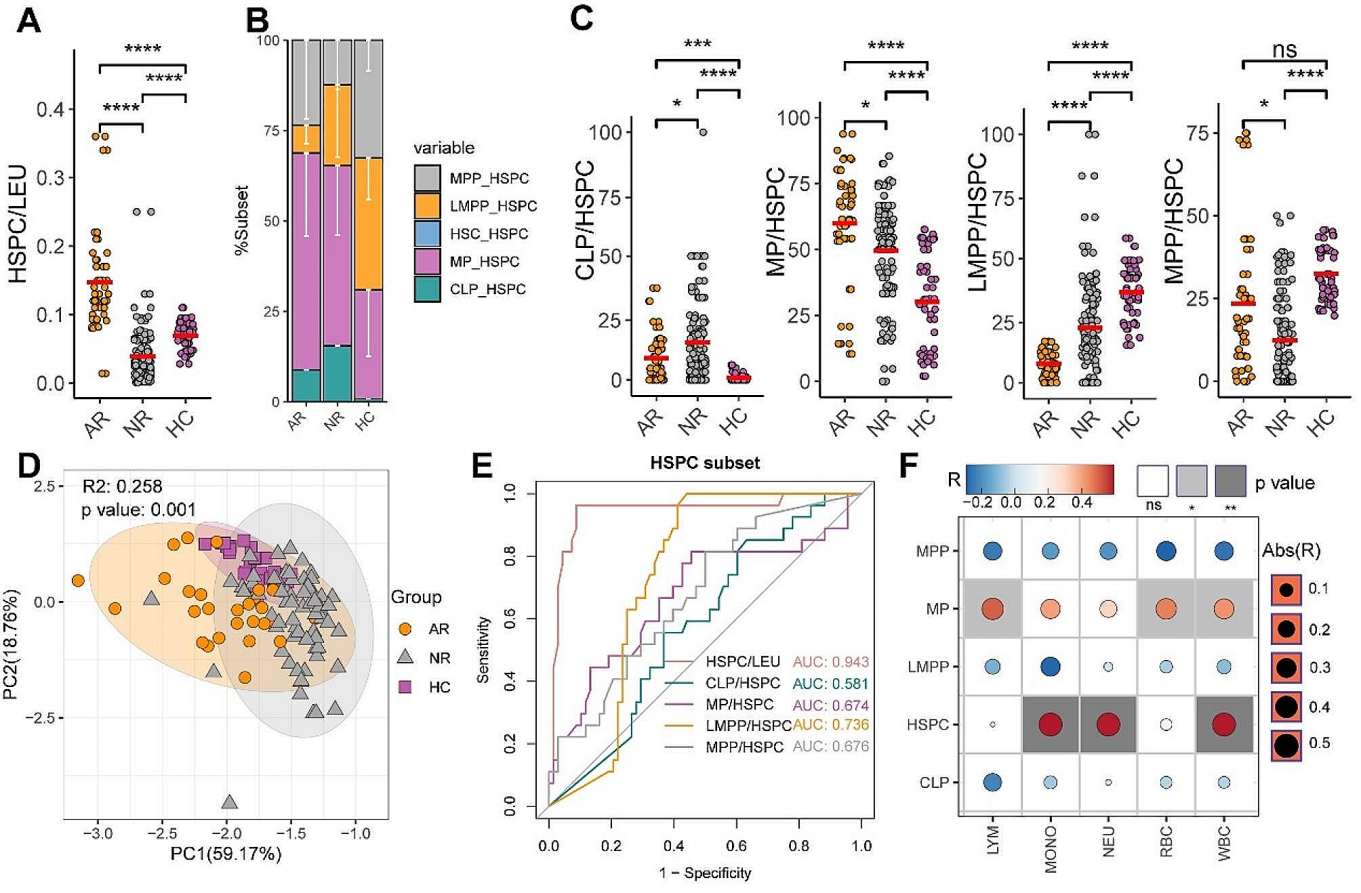
HSPCs and subsets distribution in blood of AR NR patients were different from HC before transplantation. (**A**) The percentage of HSPC in leukocytes (LEU) in the AR, NR and HC groups. (**B**) Composition of preoperative HSPC subsets in the AR, HC and NR groups. (**C**) comparison of percentage of CLP, MP, LMPP, MPP to HSPC among the AR, NR and HC groups. (**D**) PCA analysis was used to distinguish the AR, NR and HC groups with the index of HSPC subset composition. (**E**) AR prediction ability of preoperative HSPCs and their subsets index was evaluated by ROC curve with the area under the curve (AUC). (**F**) Correlation analysis of the HSPCs proportions and subpopulation composition with the absolute counts of white blood cells (WBC), lymphocytes (LYM), red blood cells (RBC), monocytes (MONO), and neutrophils (NEU) in the pre-transplant peripheral blood. R represents the correlation coefficient. The blue represents negative correlation coefficient; the red represents positive correlation coefficient. The white box indicates a Pearson test p-value greater than 0.05, gray represents the p-value less than 0.05. The size of the circle represents the absolute value of the correlation coefficient

We measured the HSPC ratio in PBMC samples collected before transplantation in the two groups of liver transplant patients and compared them with the age-matched healthy blood donors. Interestingly, we found the PBMC samples of the AR group contained a significantly higher proportion of HSPCs compared with the NR or HC groups, while samples of the NR group were lower in the HSPC proportion than the HC or AR group. The result suggests patients who develop into AR after LTx harbor more HSPCs in the blood circulation before transplant than the age-matched healthy controls, conversely the ones without rejection in the first year after transplant harbor fewer HSPCs than the healthy controls (Fig. 2A). The proportion of HSPCs in the preoperative blood of LTx patients was positively correlated with the absolute counts of white blood cells (WBC), monocytes (MONO), neutrophils (NEU) (Fig. 2F).

To further profile the subset composition of HSPCs from LTx recipients before transplant, we divided the cells into five subsets by multi-color flow cytometry (Figure S1B), including HSC, MPP, LMPP, CLP, and MP. We observed differences in the HSPC subsets distribution among the three groups: compared with the age-matched HC group, the end-stage liver disease (ESLD) patients tend to have more linage-committed CLP and MP in their blood, and have proportionally fewer LMPP and MPP subsets. Within the two LTx groups, HSPCs in PBMC samples of AR patients before transplant exhibited a higher proportion of MP and MPP composition (Fig. 2B, C), and the MP proportion was positively correlated with the absolute counts of WBC, lymphocytes (LYM), and red blood cells (RBC) (Fig. 2F). While the CLP and LMPP percentages were higher in the NR patients (Fig. 2B, C). The proportion of HSC showed no significant difference between AR, NR, and HC groups, and presented a neglectable amount in the blood HSPCs (Figure S1C).

Further analysis involved an unsupervised clustering analysis of HSPC subpopulations. The results demonstrated that these five subpopulations effectively distinguished the AR, NR, and HC groups (Fig. 2D). Notably, when using these indexes to predict AR after LTx, the total HSPC proportion in PBMC samples showed the highest predictive value (Fig. 2E).

### Donor HSPCs chimerism was found at negligible levels in recipient blood without GVHD after LTx

**Fig. 3:**
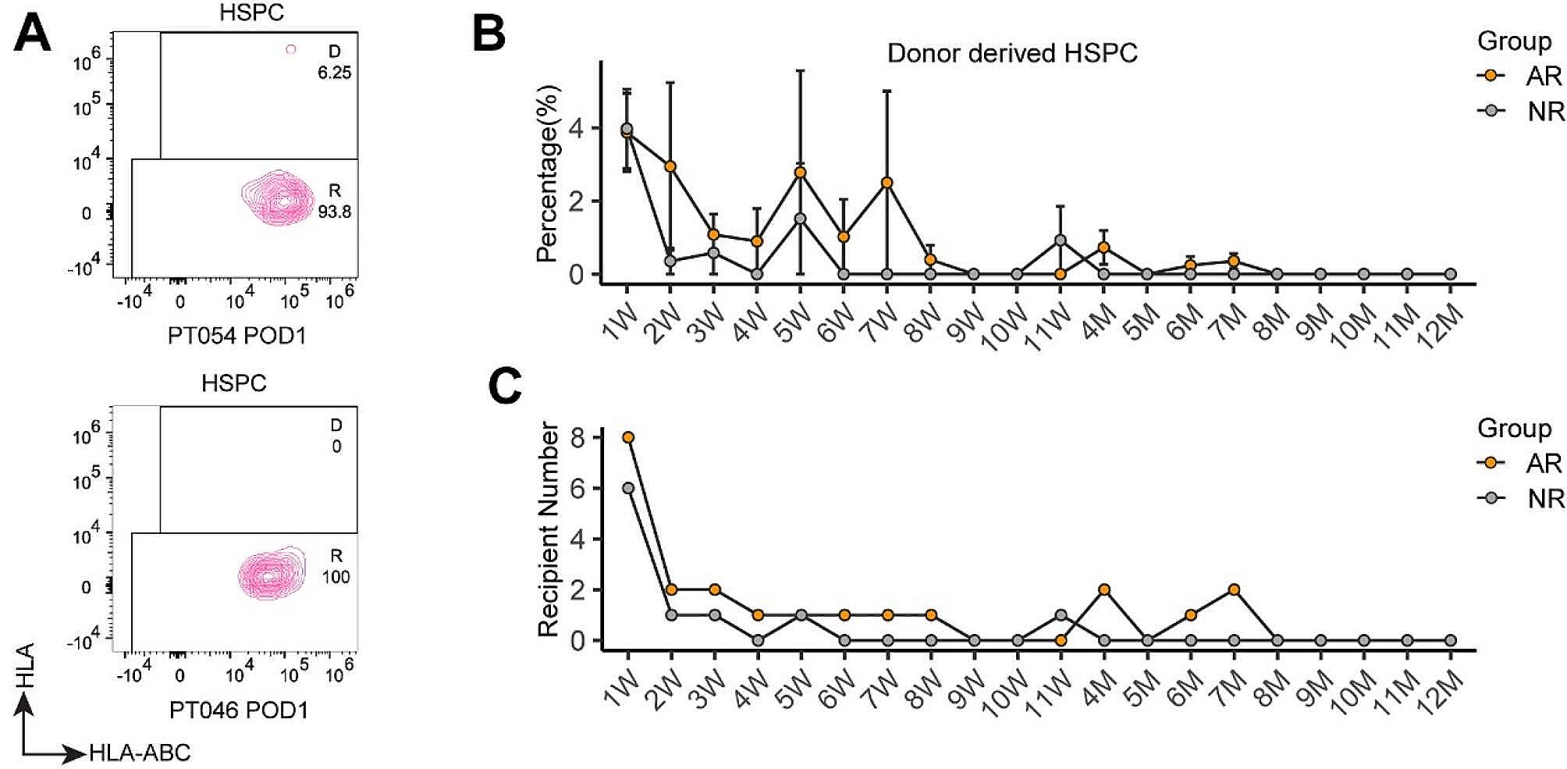
Donor HSPC chimerism quantification in recipient blood. (**A**) Gating strategy of identification of donor HSPCs in patient’s PBMC samples by MCFC, donor HSPCs were identified on the first day after surgery for PT054 with rejection episode and Pt046 without rejection episode. D represents Donor, R represents Recipient. (**B**) Donor HSPC chimerism ratio after LTx in the AR and NR group. (**C**) Number of patients whose PBMC sample contained MCFC-detectable donor HSPC after LTx in the AR group and the NR group

Donor HSPCs is believed to be involved in post-transplant immune response toward the graft [14, 15]. The human adult liver harbors HSPCs [16]. To determine if the donor HSPCs in the human liver allograft can join the host blood circulation after transplantation, we longitudinally detected the donor HSPCs by combining donor- or recipient-specific HLA antibody with a pan-HLA-ABC monoclonal antibody (mAb) by MCFC as reported [12] (Fig. 3A). All postoperative PBMC samples contained detectable levels of HSPCs, but the donor HSPCs chimerism was mainly detectable in the first two months with a ratio lower than 4% in only 13 subjects’ PBMC samples. After 2 months of LTx, PBMC samples of only two recipients in the AR group and one recipient in the NR group contain MCFC-detectable donor HSPC (Fig. 3B, C). Hence, the donor HSPCs chimerism in host blood was infrequent and was present at negligible levels after human LTx.

### HSPCs in blood of rejectors decrease in proportion rapidly after LTx

**Fig. 4:**
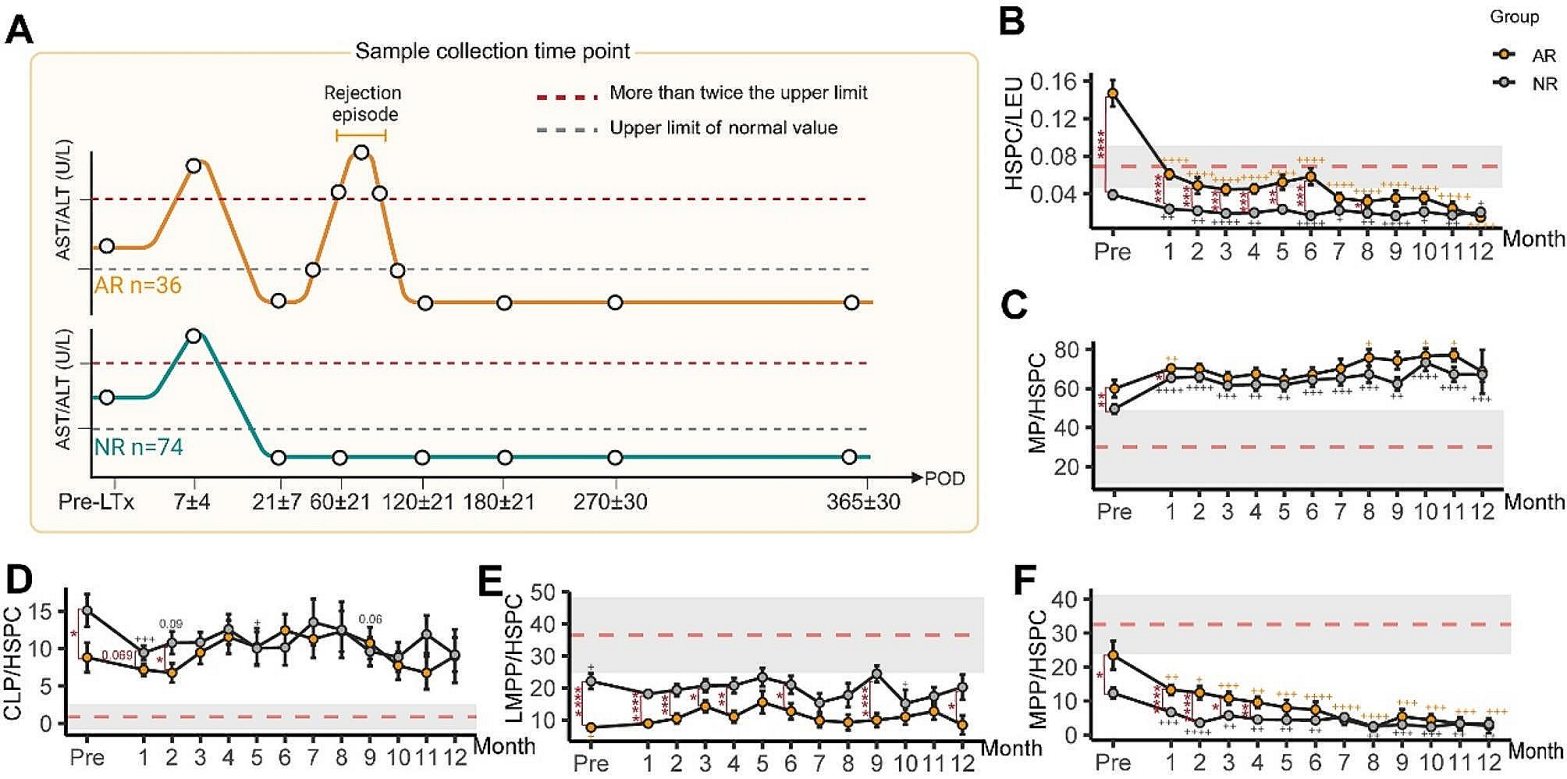
HSPCs and subset composition dynamics after LTx in AR and NR groups. (**A**) Time points for collecting blood samples of the HC, NR cohort and AR cohort. The circles in the figure represent the time points for sample collection. (**B**) The proportion of HSPC in leukocytes in PBMC samples from AR (yellow dot) and NR (grey dot) before and after LTx one-year. the proportion of MP (**C**), CLP (**D**), LMPP (**E**) and MPP (**F**) in HSPCs in recipients blood. * represents statistical differentiation between AR and NR group. + represents statistical differentiation between Pre-LTx and post-LTx timepoints in AR or NR group. * or + represents *P* < 0.05, ** or + + represents *P* < 0.01, *** or +++ represents *P* < 0.001, **** or ++++ represents *P* < 0.0001

The proportion of HSPCs and their subsets in recipients were measured in the one-year follow-up period (Fig. 4A). We found the HSPC percentage in AR patients’ blood rapidly decreased after transplantation, it continuously dropped to less than HC levels within 6 months after LTx. The proportion of HSPCs in the AR group was still significantly higher than in the NR group in the first 8 months after transplantation, but the statistical difference between the two groups disappeared afterward (Fig. 4B).

Of note, the proportion of MP in recipients’ blood HSPCs after LTx grew even higher after transplantation (Fig. 4C). In contrast, the proportion of CLP in HSPCs of LTx recipients reduced after transplant, albeit it still maintained higher than that in HC during the first year after transplantation. The preoperative significant difference in CLP between the two LTx groups gradually disappeared within the first 3 months after transplantation (Fig. 4D). As for the LMPP and MPP, they still exhibited lower proportions than the HC group after LTx, and the MPP percentage in HSPCs in AR patients grew even lower after transplantation (Fig. 4E, F).

Taken together, HSPCs and their subsets still exhibited remarkable distinction from the age-matched healthy population even after the ESLD was treated, and the high MP proportion is the feature of LTx recipient blood HSPCs after LTx.

### HSPCs mobilized in recipient blood circulation during AR

**Fig. 5:**
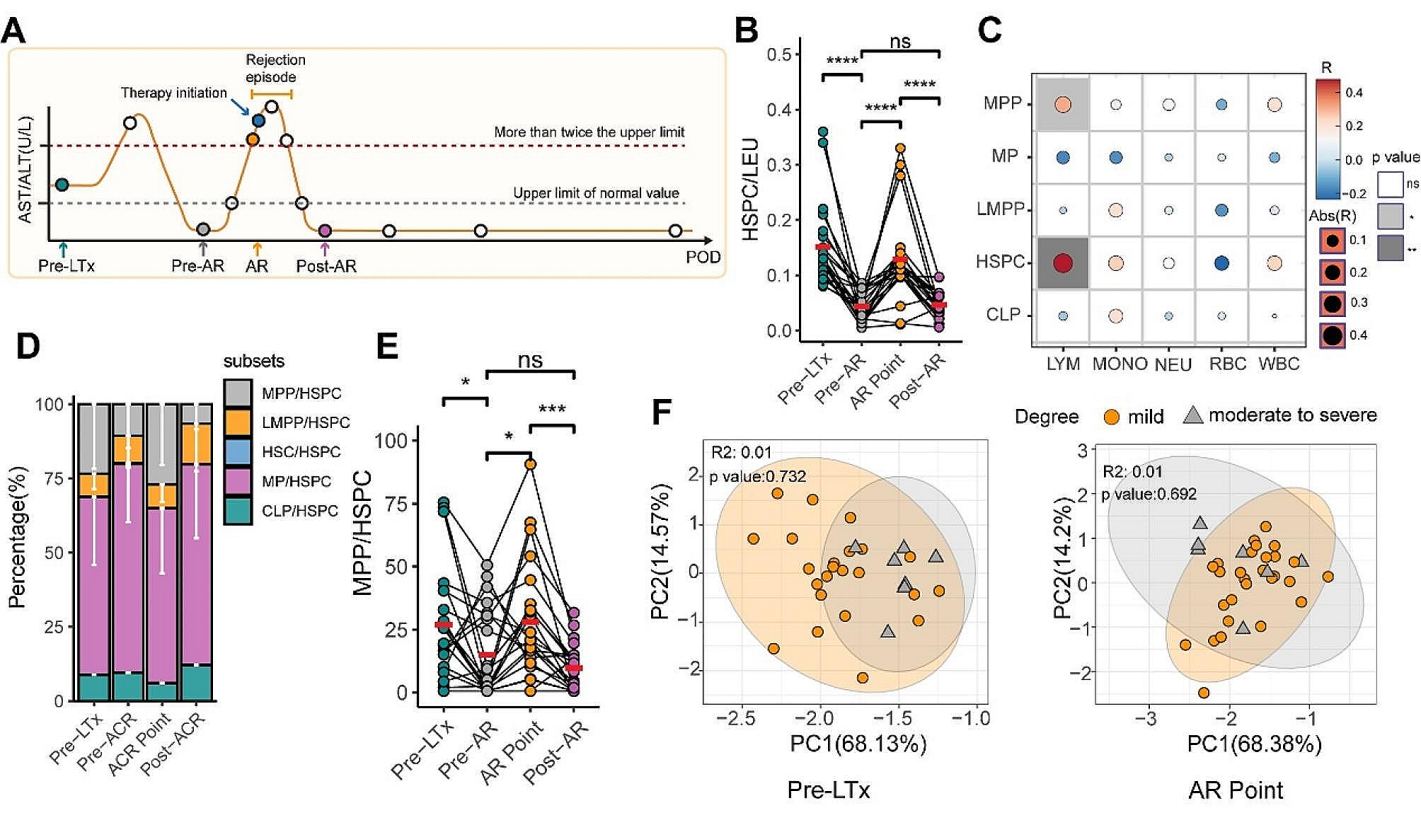
The alteration of HSPCs and subsets composition during AR episode. (**A**) The schematic diagram of analysis timepoint, pre-LTx is the timepoint before transplantation, pre-AR is the timepoint before rejection occurrence, AR is the first point of rejection occurrence and before treatment, post-AR is the first timepoint of rejection relief. **B**) Comparison of the proportion of HSPC to leukocytes at the 4 timepoints. (**C**) Correlation analysis of the proportions of HSPCs and their subpopulations in the peripheral blood of patients at AR point with the counts of WBC, LYM, RBC, MONO, RBC, and NEU. R represents the correlation coefficient. The blue represents negative correlation coefficient; the red represents positive correlation coefficient. The white box indicates a Pearson test p-value greater than 0.05, gray represents the p-value less than 0.05. The size of the circle represents the absolute value of the correlation coefficient. (**D**) Distribution of HSPC subsets at the 4 timepoints. (**E**) Comparison of the proportion of MPP in HSPC at the 4 timepoints. (**F**) Using PCA to analyze HSPCs and their subtypes to distinguish differences between patients with mild and moderate to severe levels of rejection

To investigate the changes in the HSPC and the subsets composition during an AR episode, we selected three postoperative PBMC samples of each patient in AR group based on the ALT/AST levels: samples before transaminase rising were defined as Pre-AR, samples of the first point at which transaminase exceeds twice the normal value but before anti-AR treatment was defined as AR, and samples at the first point when transaminase drops to the normal range was defined as Post-AR (Fig. 5A). To rule out the time-dependent change of HSPC after LTx, we also selected the Pre-LTx samples in the comparison.

The postoperative reduction in HSPCs proportion boosted back during AR, may suggest the AR induces HSPCs mobilizing to peripheral blood (Fig. 5B). The proportion of HSPCs was significantly positively correlated with the LYM count during AR (Fig. 5C). The high HSPC percentage was only transient in blood as it dropped after AR was relieved (Fig. 5B). Among these time points, we studied the distribution of HSPCs subsets and found that the MP subsets accounted for the largest proportion, exceeding 60% (Fig. 5D), however, they exhibited no significant variation in percentage during AR (Figure S1E). We observed that the MPPs increased during AR, and their proportion in HSPCs was significantly positively correlated with the LYM count (Fig. 5B, C), they subsequently reduced after AR episode, the tendency of which coincides with that of total HSPCs (Fig. 5E). No significant differences were observed in the proportions of HSCs, CLPs, and LMPP during the AR episode (Figure S1D, F, G).

To determine if the recipient-derived HSPCs can migrate into the liver allograft during rejection, HMC were collected from an explanted liver allograft of a patient who undergone secondary LTx because of developing into chronic rejection after AR. In the one case, we found donor HSPCs in the pre-transplant liver allograft as expected, 70% of the HSPCs are multipotent stem and progenitor cells (HSC, MPP, LMPP) (Figure S1H, J). While at the secondary transplant time point, most of the HSPCs were lineage-committed progenitor (CLP and MP), and all MPP and nearly half of MP were replaced by recipient cells (Figure S1I, K).

To determine if the HSPC and subsets proportion in blood correlated with the severity of rejection, AR patients were assigned into mild rejection group (*n* = 29) and moderate to severe rejection group (*n* = 7). No significant difference between the two groups were observed at the timepoints of pre-LTx and AR points in this study, the unsupervised clustering analysis of HSPC subpopulations also suggested no significant difference between the two groups (Fig. 5F).

Together, recipients HSPCs mobilized to the blood-circulation and migrated to the liver allograft during AR.

### Donor HSPCs mobilized in the blood of recipient with post-LTx GVHD

**Fig. 6:**
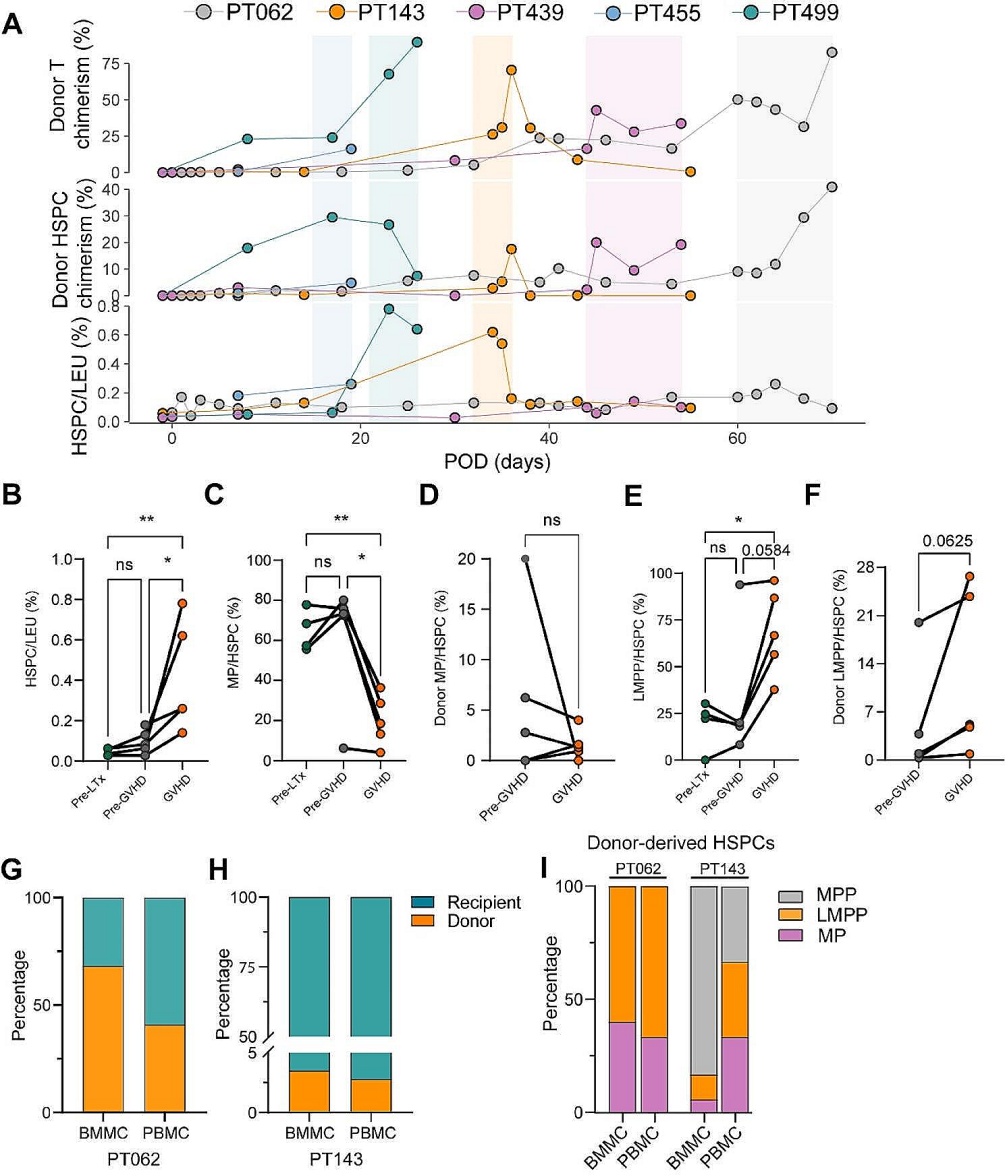
The dynamics of HSPCs and subsets composition during GVHD episode. (**A**) Dynamics of donor T cells chimerism ratios (upper), donor HSPCs chimerism ratios (middle), and total HSPC to leukocyte ratio (lower). Each patient’s data was present in one color, and the block represents a GVHD episode of each patient. The donor T cells chimerism ratios of PT62 and PT143 have been reported in our previous study [1]. (**B**) The ratio of HSPC in pre-LTx, pre-GVHD, GVHD. (**C**)The ratio of MP in pre-LTx, pre-GVHD, GVHD (**D**) The proportion of donor-derived MP in pre-GVHD, GVHD. (**E**) The ratio of LMPP in pre-LTx, pre-GVHD, GVHD. (**F**) The proportion of donor-derived LMPP in pre-GVHD, GVHD. The chimerism ratio of donor-derived HSPCs in the bone marrow of (**G**) PT062 and (**H**) PT143. (**I**) The composition of donor-derived HSPC subtypes in peripheral blood and bone marrow sampled on the same day from PT062 and PT143

To investigate the dynamics of HSPCs in the rejection toward the recipient, five LTx recipients with post-LTx GVHD were enrolled to perform a longitudinal analysis of HSPCs and their subsets in PBMCs before and during the GVHD episode. As aforementioned AR results, we also found the HSPCs accumulated in the recipient’s blood circulation during the GVHD episode. By determining the cell origin using donor recipient-distinguish MHC antibodies, we found the donor-derived HSPCs heavily accumulated in recipients’ blood during or even before the disease onset, which is in contrast to negligible levels of donor HSPCs chimerism in the rejectors. The donor HSPCs chimerism ratio was synchronized with the ratio of donor T cells (Fig. 6A, B). The proportion of MP subsets decreased dramatically during GVHD (Fig. 6C). The similar tendency was observed in the ratio of donor-derived MP (Fig. 6D). The proportion of LMPP subsets and the donor LMPP chimerism ratio showed an increased tendency at the onset of GVHD (Fig. 6E, F), reflecting the lymphocyte-biased differentiation during GVHD.

To determine if the donor-derived HSPCs within the liver allograft can migrate to recipient’s target tissue, BMMC samples from PT62 and PT143 collected during GVHD were analyzed. In the two cases, we detected considerable amount of donor-derived HSPCs in recipient’s bone marrow, and the ratio of donor-derived HSPCs in the bone marrow is greater than that in peripheral blood (PT62: BMMC 68.2% vs. PBMC 40.9%, PT143 BMMC 3.5% vs. PBMC 2.8%) (Fig. 6G, H), and all the donor HSPC subsets presented in blood can be found in the bone marrow (Fig. 6I).

Together, donor HSPCs in the liver allograft mobilized to the blood-circulation and migrated to the recipient’s bone marrow during GVHD.

## Discussion

Alloresponse has a detrimental effect on long-term graft survival [17, 18], where mature immune cells like T cells have been described as key players. An increase in the proportion of immature immune cells in the recipient’s circulation during AR has also been reported [19], hinting an emergency myelopoiesis may also exist during the alloresponse. However, HSPCs differentiation pattern and mobilization dynamics in the alloimmune response remain unexplored.

In patients with ESLD, we demonstrated that a higher proportion of HSPCs was present in the preoperative PBMC of recipients who would develop AR after transplantation, it would reflect a younger age of the AR group [20], and may also suggest that these patients were more active in hematopoiesis as HSPC proportion showed correlation with the immune cell absolute counts. Through further analysis, we revealed that these HSPCs skewed toward producing MP subsets. Such bias might indicate emergency myelopoiesis in patients with end-stage liver failure to meet the increased demand for innate immune cells [21] or indicate an immunosenescent state of the HSPCs from those patients who persist in inflammation [22, 23].

After transplantation, HSPCs rapidly declined in blood and their subsets composition were still significantly different from healthy controls, the MP-biased differentiation became even more remarkable. Previous studies reported that immunosuppressants, such as sirolimus, block the CXCR4-CXCL12 axis that regulates HSPCs migration and homing [24]. The postoperative HSPCs proportion decline in blood in both AR and NR groups of transplant recipients is possibly caused by immunosuppression therapy. The MP-biased differentiation may be also caused by the immunosuppression therapy since immunosenescence signs were reported in patients with long term or enhanced immunosuppression therapy [25, 26].

The formation of chimerism after transplantation is closely related to alloimmune response [12, 27]. However, only macrochimerism in mature cells was investaged [28, 29]. In this study, we observed negligible levels of donor HSPC chimerism in AR and stable recipients who with negligible donor T cell chimerism ratios [30]. HSPCs have been found in some non-lymphoid tissues in adult humans, such as kidney [15], intestine [31], and liver [32]. In the context of organ transplantation, those HSPCs are transferred to recipients. We found remarkable amount of donor HSPC in the blood circulation and bone marrow of patients with GVHD, the ratio coincided with that of donor T cells, aligned with the study in intestinal transplantation [14], indicating the chimerism of donor HSPC is a result of donor T cells mediated GVH reaction.

In the case of infection, injury, and stress, HSPCs migrate to the damaged tissues through circulation to replenish mature immune cells [33, 34]. In the case of alloresponse, we found that the recipient- and donor-derived HSPCs were mobilized rapidly into blood-circulation during AR and GVHD, respectively, and they may reach to the target tissue. Expression of stromal cell-derived factor-1 (SDF-1) in the liver and elevated level of matrix metalloproteinases 9 (MMP9) in the serum during rejection would significantly contribute to the HSPCs migration and mobilization [35]. MMP-9 is known for its ability to degrade extracellular matrix components, which is crucial for the mobilization of HSPCs by remodeling of the bone marrow niche [36–39]. Additionally, MMP-9 can cleave and activate various cytokines and chemokines, further enhancing the inflammatory response and facilitating the migration of HSPCs to the target tissues [40]. SDF-1 expressed on the bile ducts in portal tracts of the rejected liver creating a chemotactic gradient that directs the migration of HSPCs through the interaction with its receptor CXCR4 to the liver [41–43].

MP and MPP represented the dominant recipient HSPCs in blood during AR and in the rejected liver, highlighted a role of recipient-derived emergency myelopoiesis in AR following LTx. Pro-inflammatory cytokines, such as IL-6, TNF-α, and IL-1β, promote myelopoiesis by enhancing the proliferation and differentiation of MP [44]. Biliary epithelial cells (BECs), the major producer of SDF-1 in the rejected liver, upregulate the production of these pro-inflammatory cytokines during inflammation [45, 46], and secrete chemokines CXCL12 and CCL2 [47] to mediate the retention of HSPCs, thereby could be the significant promotor of emergency myelopoiesis during LTx AR. Previous reports [16, 48, 49] and our unpublished data evidenced that healthy adult human liver harbor multipotent stem and progenitor cells (HSC, MPP, LMPP) and committed precursors (CLP, MP), their ability of bone marrow homing was indirectly verified by a case report of full hematologic chimerism in a post-LTx patient [50]. In this study, we directly showed that donor multipotent stem and progenitor cells and committed precursors in the liver allograft can home to recipient bone marrow niche. However, their migration to target tissue during GVHD still needs investigation in other type of target tissue other than the bone marrow niche.

It is a single center study with limited sample size, patients in the study cohort with high HBV-dominated primary disease which is different from other parts of the world. The result needs to be further corroborated by other regions of the world. Due to the limitation of detection techniques, insufficient number of HSPCs in liver biopsy samples for analysis, and only one rejected liver allograft sample from a secondary LTx in this study, research should be carried out in further to characterize the recipient HSPCs in rejected livers.

This study demonstrates the characterization of HSPCs in the blood circulation of patients pre- and post-LTx and describes the mobilization during LTx rejection. Our results provide a basis for in-depth studies on the role of HSPC in promoting and predicting rejection after LTx in humans.

## Conclusions

The AR group had a higher proportion of HSPCs in pre-transplant blood compared to the NR and HC groups, indicating a potential predictive marker for AR. Post-transplant, HSPC levels in AR patients rapidly decreased, and all patients showed MP-biased differentiation. Recipient- and donor-derived HSPCs mobilized in blood during AR and GVHD and migrated to target tissues, respectively. These findings underscore the role of HSPCs in transplant outcomes and potential implications for monitoring and managing liver transplant patients.

### Electronic supplementary material

Below is the link to the electronic supplementary material.

Supplementary Material 1

## Data Availability

Data will be available on reasonable request.

## Abbreviations

AR: Acute rejection
BMMC: Bone marrow mononuclear cell
CLPs: Common lymphoid progenitors
ESLD: End-stage liver disease
GVHD: Graft-versus-host disease
HC: Healthy control
HLA: Human leukocyte antigen
HMC: Hepatic mononuclear cell
HSCs: Hematopoietic stem cells
HSPCs: Hematopoietic stem and progenitor cells
LMPPs: Lymphoid-primed multipotent progenitors
LTx: Liver transplantation
LYM: Lymphoncyte
mAb: Monoclonal antibody
MCFC: Multi-color flow cytometry
MHC: Major histocompatibility complex
MONO: Monocyte
MPPs: Multi-potent progenitors
MPs: Myeloid progenitors
NEU: Neutriphils
NR: Recipients without rejection
PBMC: Peripheral blood mononuclear cell
PBS: Phosphate-buffered saline
POD: Postoperative day
RBC: Red blood cells
ROC: Receiver operating characteristic curve
WBC: White blood cells
