# Supplementary material for: Donor and recipient hematopoietic stem and progenitor cells mobilization in liver transplantation patients
Source: Stem Cell Res Ther. 2024 Jul 29;15:231. doi: 10.1186/s13287-024-03855-5 (PMC11288126; doi:10.1186/s13287-024-03855-5)
Supplement: Supplementary file 1 — Supplementary Material 1 [file 13287_2024_3855_MOESM1_ESM.docx]

Supplementary Materials for

**Donor and recipient hematopoietic stem and progenitor cells mobilization in liver transplantation patients**

Yao Zhi, Wei Qiu, Guangyao Tian, Shifei Song, Wenchao Zhao, Xiaodong Du, Xiaodong Sun, Yuguo Chen, Heyu Huang, Jing Li, Ying Yu, Mingqian Li*, Guoyue Lv*

**Affiliations:** Department of Hepatobiliary and Pancreatic Surgery, General Surgery Center, The First Hospital of Jilin University, Changchun 130021, China


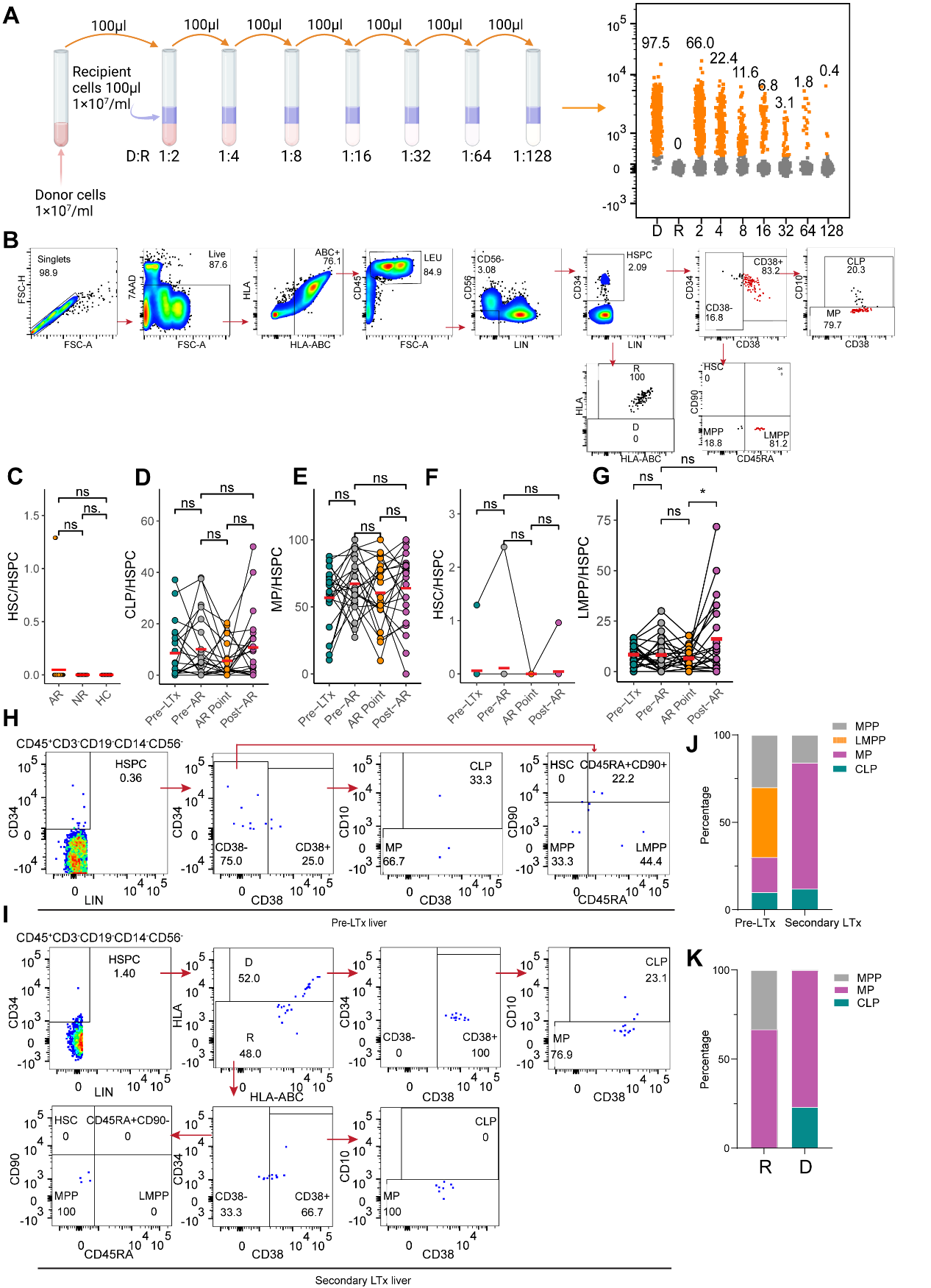


**Supplementary figure 1:** (A) Flowchart for the donor HLA mAb sensitivity assay. (B) The schematic diagram of representative flow cytometry gate strategy of HSPC phenotype and identification of donor and recipient HSPC. (C) Comparison of hematopoietic stem cells (HSC) among AR, NR and HC groups. Comparison of CLP (D), MP (E), HSC (F) and LMPP (G) percentage in HSPCs in blood at selected timepoints in AR group. (H) Flow cytometry analysis of HSPCs and their subtypes in the pre-transplant liver tissue and (I) rejected liver tissue. D represents the donor, and R represents the recipient. (J) Stacked bar chart of HSPCs subtypes from pretransplant liver and rejected liver. (K) Stacked bar chart of recipient- and donor-derived HSPCs subtypes from rejected liver.

**Supplementary table 1: Antibody list**

| Antibodies | Clone | Source | Identifier |
| --- | --- | --- | --- |
| 7AAD | NA | BD Pharmingen | Cat#559925;  RRID:AB_2869266 |
| Anti-Human HLA-ABC | W6/32 | Biolegend | Cat# 311436;  RRID: AB_2566255 |
| Anti-Human HLA-A2 FITC | BB7.2 | BD Pharmingen | Cat# 551285;  RRID: AB_394130 |
| Anti-Human HLA-A3 FITC | REA950 | Miltenyi Biotec | Cat# 130-115-739;  RRID: AB_2727169 |
| Anti-Human HLA-A9 FITC | REA127 | Miltenyi Biotec | Cat# 130-099-524;  RRID: AB_2652070 |
| Anti-Human HLA-A11 Biotin | BIH0084 | One Lambda | Cat# BIH0084;  RRID:NA |
| Anti-Human HLA-BW4 FITC | REA274 | Miltenyi Biotec | Cat#130-103-846;  RRID: AB_2652010 |
| Anti-Human HLA-BW6 FITC | REA143 | Miltenyi Biotec | Cat# 130-123-264;  RRID: AB_2819460 |
| FITC streptavidin | NA | Biolegend | Cat# 405202;  RRID:NA |
| Anti-Human CD45 PE-Cy7 | HI30 | Invitrogen | Cat# 25-0459-42;  RRID: AB_1944375 |
| Anti-Human CD56 SB600 | TULY56 | Invitrogen | Cat#63-0566-42;  RRID:AB_2662561 |
| Anti-Human CD3 APC-Cy7 | SK7 | BD Pharmingen | Cat# 560176;  RRID: AB_1645475 |
| Anti-Human CD19 APC-Cy7 | HIB19 | Biolegend | Cat# 302218;  RRID: AB_314248 |
| Anti-Human CD14 APC-Cy7 | M5E2 | Biolegend | Cat# 301820;  RRID: AB_493695 |
| Anti-Huma CD34 PE | QBEND/10 | Invitrogen | Cat#MA1-10205;  RRID: AB_11152571 |
| Anti-Huma CD38 AF700 | HIT2 | BD Horizon | Cat# 564979;  RRID: AB_2744373 |
| Anti-Huma CD10 SB436 | eBioCB-CALLA | Invitrogen | Cat# 62-0106-42;  RRID: AB_2716983 |
| Anti-Huma CD90 APC | 5E10 | Biolegend | Cat# 328114;  RRID: AB_893431 |
| Anti-Huma CD45RA PE-CF594 | HI100 | BD Horizon | Cat# 562298;  RRID: AB_11154413 |
